# flowClust: a Bioconductor package for automated gating of flow cytometry data

**DOI:** 10.1186/1471-2105-10-145

**Published:** 2009-05-14

**Authors:** Kenneth Lo, Florian Hahne, Ryan R Brinkman, Raphael Gottardo

**Affiliations:** 1 Department of Statistics, University of British Columbia, 333-6356 Agricultural Road, Vancouver, BC, V6T1Z2, Canada; 2 Fred Hutchinson Cancer Research Center, 1100 Fairview Avenue North, Seattle, WA 98109, USA; 3 Terry Fox Laboratory, BC Cancer Research Center, 675 West 10th Avenue, Vancouver, BC, V5Z1L3, Canada; 4 Institut de recherches cliniques de Montreal, 110, avenue des Pins Ouest, Montreal, QC, H2W 1R7, Canada; 5 Département de biochimie, Université de Montreal, 2900, boul Edouard-Montpetit, Montreal, QC, H3T 1J4, Canada

## Abstract

**Background:**

As a high-throughput technology that offers rapid quantification of multidimensional characteristics for millions of cells, flow cytometry (FCM) is widely used in health research, medical diagnosis and treatment, and vaccine development. Nevertheless, there is an increasing concern about the lack of appropriate software tools to provide an automated analysis platform to parallelize the high-throughput data-generation platform. Currently, to a large extent, FCM data analysis relies on the manual selection of sequential regions in 2-D graphical projections to extract the cell populations of interest. This is a time-consuming task that ignores the high-dimensionality of FCM data.

**Results:**

In view of the aforementioned issues, we have developed an **R **package called **flowClust **to automate FCM analysis. **flowClust **implements a robust model-based clustering approach based on multivariate *t *mixture models with the Box-Cox transformation. The package provides the functionality to identify cell populations whilst simultaneously handling the commonly encountered issues of outlier identification and data transformation. It offers various tools to summarize and visualize a wealth of features of the clustering results. In addition, to ensure its convenience of use, **flowClust **has been adapted for the current FCM data format, and integrated with existing Bioconductor packages dedicated to FCM analysis.

**Conclusion:**

**flowClust **addresses the issue of a dearth of software that helps automate FCM analysis with a sound theoretical foundation. It tends to give reproducible results, and helps reduce the significant subjectivity and human time cost encountered in FCM analysis. The package contributes to the cytometry community by offering an efficient, automated analysis platform which facilitates the active, ongoing technological advancement.

## Background

Flow cytometry (FCM) is a high-throughput technology that offers rapid quantification of a set of physical and chemical characteristics for a large number of cells in a sample. FCM is widely used in health research and treatment for a variety of tasks, such as providing the counts of helper-T lymphocytes needed to monitor the course and treatment of HIV infection, in the diagnosis and monitoring of leukemia and lymphoma patients, the evaluation of peripheral blood hematopoietic stem cell grafts, and many other diseases [1-8]. The technology is also used in cross-matching organs for transplantation and in research involving stem cells, vaccine development, apoptosis, phagocytosis, and a wide range of cellular properties including phenotype, cytokine expression, and cell-cycle status [9-14].

Currently, FCM can be applied to analyze thousands of samples per day. Nevertheless, despite its widespread use, FCM has not reached its full potential due to the lack of an automated analysis platform to parallel the high-throughput data-generation platform. In contrast to the tremendous interest in the FCM technology, there is a dearth of statistical and bioinformatics tools to manage, analyze, present, and disseminate FCM data. There is considerable demand for the development of appropriate software tools, as manual analysis of individual samples is error-prone, non-reproducible, non-standardized, not open to re-evaluation, and requires an inordinate amount of time, making it a limiting aspect of the technology [1,7,15-21].

One core component of FCM analysis involves gating, the process of identifying cell populations that share a set of common properties or display a particular biological function. Currently, to a large extent, gating relies on the sequential application of a series of manually drawn gates (i.e., data filters) that define regions in 1- or 2-D graphical projections of FCM data. This process is manually time-consuming and subjective as researchers have traditionally relied on intuition rather than standardized statistical inference [7,22,23]. In addition, this process ignores the high-dimensionality of FCM data, which may convey more information than that provided by only looking at 1- or 2-D projections.

Recently, a suite of several **R **packages providing infrastructure for FCM analysis have been released though Bioconductor [24], an open source software development project for the analysis of genomic data. **flowCore **[25], the core package among them, provides data structures and basic manipulation of FCM data. **flowViz **[26] offers visualization tools, while **flowQ **provides quality control and quality assessment tools for FCM data. Finally, **flowUtils **provides utilities to deal with data import/export for **flowCore**. In spite of these low-level tools, there is still a dearth of software that helps automate FCM gating analysis with a sound theoretical foundation [15].

In view of these issues, based on a formal statistical clustering approach, we have developed the **flowClust **package (Additional file 1) to help resolve the current bottleneck. **flowClust **implements a robust model-based clustering approach [27-29] which extends the multivariate *t *mixture model with the Box-Cox transformation originally proposed in [30]. As a result of the extensions made, **flowClust **has included options allowing for a cluster-specific estimation of the Box-Cox transformation parameter and/or the degrees of freedom parameter; the Implementation section and the Results and Discussion section provide a detailed account of these extensions.

## Implementation

### The model

In statistics, model-based clustering [28,29,31,32] is a popular unsupervised approach to look for homogeneous groups of observations. The most commonly used model-based clustering approach is based on finite Gaussian mixture models, which have been shown to give good results in various applied fields [28,29,33,34]. However, Gaussian mixture models might give poor representations of clusters in the presence of outliers, or when the clusters are far from elliptical in shape, phenomena commonly observed in FCM data. In view of this, we have proposed in [30] an approach based on *t *mixture models [27,28] with the Box-Cox transformation to handle these two issues simultaneously. Formally, given independent *p*-dimensional multivariate observations **y**_1_, **y**_2_,...,**y**_*n*_, and denoting by *Ψ *the collection of all unknown parameters, the likelihood for a mixture model with *G *components is

(1)

where *w*_*g *_is the probability that an observation belongs to the *g*-th component, and *φ*_*p*_(·|***μ***_*g*_, **Σ**_*g*_, *ν*_*g*_) is the *p*-dimensional multivariate *t *density with mean ***μ***_*g *_(*ν*_*g *_> 1), covariance matrix *ν*_*g *_(*ν*_*g *_- 2)^-1 ^**Σ**_*g *_(*ν*_*g *_> 2) and *ν*_*g *_degrees of freedom.  is the value obtained upon transforming **y**_*i *_with the Box-Cox parameter *λ*_*g*_; the transformation used is a variant of the original Box-Cox transformation which is also defined for negative-valued data [35]. Finally,  is the Jacobian induced by the transformation. Please refer to [30] for a detailed account of an Expectation-Maximization (EM) algorithm [36] for the simultaneous estimation of all unknown parameters **Ψ **= (**Ψ**_1_,...,**Ψ**_*G*_) where **Ψ**_*g *_= (*w*_*g*_, ***μ***_*g*_, **Σ**_*g*_, *ν*_*g*_, *λ*_*g*_).

The EM algorithm needs to be initialized. By default, random partitioning is performed 10 times in parallel, and the one delivering the highest likelihood value after a few EM runs will be selected as the initial configuration for the eventual EM algorithm.

Note that, in the model originally proposed in [30], the Box-Cox parameter *λ *is set common to all components of the mixture, and the degrees of freedom parameter *ν *is fixed at a predetermined common value. In the latest development of our software, we have generalized the model such that *ν *may also be estimated, and both *λ *and *ν *are allowed to be component-specific, as reflected in Equation (1).

When the number of clusters is unknown, we use the Bayesian Information Criterion (BIC) [37], which gives good results in the context of mixture models [29,38].

### The package

With the aforementioned theoretical basis, we have developed **flowClust**, an **R **package to conduct an automated FCM gating analysis and produce visualizations for the results. Its source code is written in C for optimal utilization of system resources and makes use of the Basic Linear Algebra Subprograms (BLAS) library, which facilitates multithreaded processes when an optimized library is provided.

**flowClust **is released through Bioconductor [24], along with those **R **packages mentioned in the Background section. The GNU Scientific Library (GSL) is needed for successful installation of **flowClust**. Please refer to the vignette that comes with **flowClust **for details about installation; Windows users may also consult the README file included in the package for procedures of linking GSL to **R**.

The package adopts a formal object-oriented programming discipline, making use of the S4 system [39] to define classes and methods. The core function, flowClust, implements the clustering methodology and returns an object of class flowClust. A flowClust object stores essential information related to the clustering result which can be retrieved through various methods such as summary, Map, getEstimates, etc. To visualize the clustering results, the plot and hist methods can be applied to produce scatterplots, contour/image plots and histograms.

To enhance communications with other Bioconductor packages designed for the cytometry community, **flowClust **has been built with the aim of being highly integrated with **flowCore**. Methods in **flowClust **can be directly applied on a flowFrame, the standard **R **implementation of a Flow Cytometry Standard (FCS) file defined in **flowCore**; FCS is the typical storage mode for FCM data. Another step towards integration is to overload basic filtering methods defined in **flowCore **(e.g., filter, %in%, Subset and split) in order to provide similar functionality for classes defined in **flowClust**.

## Results and discussion

### Analysis of real FCM data

In this section, we illustrate how to use **flowClust **to conduct an automated gating analysis of real FCM data. For demonstration, we use the graft-versus-host disease (GvHD) data (Additional file 2) [40]. The data are stored in FCS files, and consist of measurements of four fluorescently conjugated antibodies, namely, anti-CD4, anti-CD8*β*, anti-CD3 and anti-CD8, in addition to the forward scatter and sideward scatter parameters. One objective of the gating analysis is to look for the CD3^+^CD4^+^CD8*β*^+ ^cell population, a distinctive feature found in GvHD-positive samples. We have adopted a two-stage strategy [30]: we first cluster the data by using the two scatter parameters to identify basic cell populations, and then perform clustering on the population of interest using all fluorescence parameters.

At the initial stage, we extract the lymphocyte population using the forward scatter (FSC-H) and sideward scatter (SSC-H) parameters:

GvHD <- read.FCS("B07", trans = FALSE)

res1 <- flowClust(GvHD, varNames = c("FSC-H", "SSC-H"), K = 1:8)

**Figure 1:**
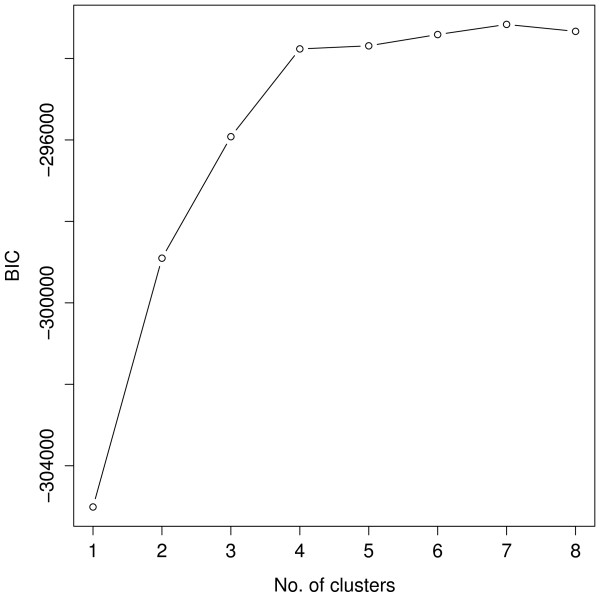
**A plot of BIC against the number of clusters for the first-stage cluster analysis**. The BIC curve remains relatively flat beyond four clusters, suggesting that the model fit using four clusters is appropriate.

To estimate the number of clusters, we run flowClust on the data repetitively with K = 1 up to K = 8 clusters in turn, and apply the BIC to guide the choice. Values of the BIC can be retrieved through the criterion method. Figure 1 shows that the BIC curve remains relatively flat beyond four clusters. We therefore choose the model with four clusters. Below is a summary of the corresponding clustering result.

** Experiment Information **

Experiment name: Flow Experiment

Variables used: FSC-H SSC-H

** Clustering Summary **

Number of clusters: 4

Proportions: 0.1779686 0.1622115 0.3882043 0.2716157

** Transformation Parameter **

lambda: 0.1126388

** Information Criteria **

Log likelihood: -146769.5

BIC: -293765.9

ICL: -300546.2

** Data Quality **

Number of points filtered from above: 168 (1.31%)

Number of points filtered from below: 0 (0%)

Rule of identifying outliers: 90% quantile

Number of outliers: 506 (3.93%)

Uncertainty summary:

Min.       1st Qu.   Median    Mean      3rd Qu.   Max.      NA's

9.941e-04 1.211e-02 3.512e-02 8.787e-02 1.070e-01 6.531e-01 1.680e+02

The estimate of the Box-Cox parameter *λ *is 0.11, implying a transformation close to a logarithmic one (*λ *= 0).

Note that, by default, flowClust selects the same transformation for all clusters. We have also enabled the option of estimating the Box-Cox parameter *λ *for each cluster. For instance, if a user finds the shapes of the clusters significantly deviate from one another and opts for a different transformation for each cluster, he may write the following line of code:

flowClust(GvHD, varNames = c("FSC-H", "SSC-H"), K = 4, trans = 2)

The trans argument acts as a switch to govern how *λ *is handled: fixed at a predetermined value (trans = 0), estimated and set common to all clusters (trans = 1), or estimated for each cluster (trans = 2). Incidentally, the option of estimating the degrees of freedom parameter *ν *has also been made available, either common to all clusters or specific to each of them. The nu.est argument is the corresponding switch and takes a similar interpretation to trans. Such an option of estimating *ν *further fine-tunes the model-fitting process such that the fitted model can reflect the data-specific level of abundance of outliers. To compare the models adopting a different combination of these options, one may make use of the BIC again. See Additional file 3 for a graph with two BIC curves corresponding to the default setting (common *λ*) and the setting with cluster-specific *λ*, respectively. Little difference in the BIC values between the two settings can be observed. In accordance with the principle of parsimony in Statistics which favors a simpler model, we opt for the default setting here.

**Figure 2:**
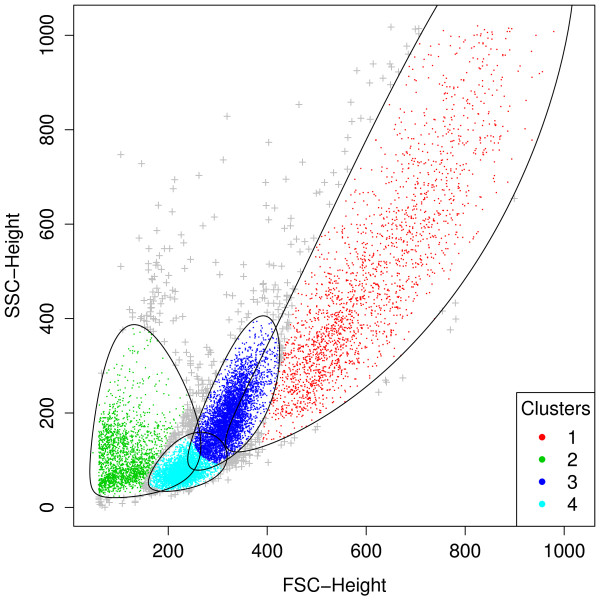
**A scatterplot revealing the cluster assignment in the first-stage analysis**. Clusters 1, 3 and 4 correspond to the lymphocyte population, while cluster 2 is referred to as the dead cell population. The black solid lines represent the 90% quantile region of the clusters which define the cluster boundaries. Points outside the boundary of the cluster to which they are assigned are called outliers and marked with "+".

Graphical functionalities are available to users for visualizing a wealth of features of the clustering results, including the cluster assignment, outliers, and the size and shape of the clusters. Figure 2 is a scatterplot showing the cluster assignment of points upon the removal of outliers. Outliers are shown in grey with the + symbols. The black solid lines represent the 90% quantile region of the clusters which defines the cluster boundaries. The summary shown above states that the default rule used to identify outliers is 90% quantile, which means that a point outside the 90% quantile region of the cluster to which it is assigned will be called an outlier. In most applications, the default rule should be appropriate for identifying outliers. In case a user wants finer control and would like to specify a different rule, he may apply the ruleOutliers replacement method:

ruleOutliers(res1[[4]]) <- list(level = 0.95)

See Additional file 4 for the corresponding summary. As shown in the summary, this rule is more stringent than the 90% quantile rule: 133 points (1.03%) are now called outliers, as opposed to 506 points (3.93%) in the default rule.

Clusters 1, 3 and 4 in Figure 2 correspond to the lymphocyte population defined with a manual gating strategy adopted in [40]. We then extract these three clusters to proceed with the second-stage analysis:

GvHD2 <- split(GvHD, res1[[4]], population = list(lymphocyte = c(1,3,4), deadcells = 2))

The subsetting method split allows us to split the data into several flowFrames representing the different cell populations. To extract the lymphocyte population (clusters 1, 3 and 4), we may type GvHD2$lymphocyte or GvHD2[[1]], which is a flowFrame. By default, split removes outliers upon extraction. The deadcells = 2 list element is included above for demonstration purpose; it is needed only if we want to extract the dead cell population (cluster 2), too.

In the second-stage analysis, in order to fully utilize the multidimensionality of FCM data we cluster the lymphocyte population using all the four fluorescence parameters, namely, anti-CD4 (FL1-H), anti-CD8*β *(FL2-H), anti-CD3 (FL3-H) and anti-CD8 (FL4-H), at once:

res2 <- flowClust(GvHD2$lymphocyte, varNames = c("FL1-H", "FL2-H", "FL3-H", "FL4-H"), K = 1:15)

**Figure 3:**
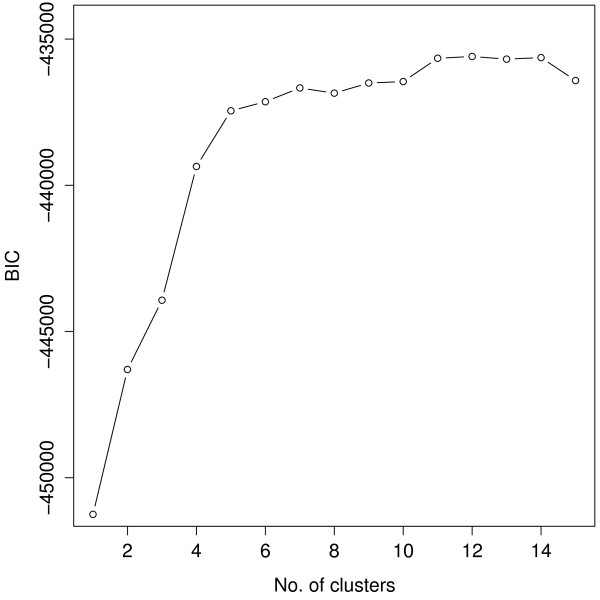
**A plot of BIC against the number of clusters for the second-stage cluster analysis**. The BIC curve remains relatively flat beyond 11 clusters, suggesting that the model fit using 11 clusters is appropriate.

**Figure 4:**
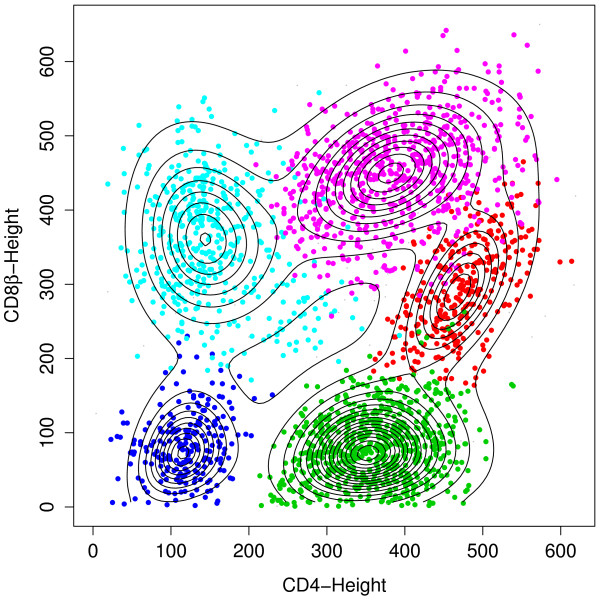
**A contour plot superimposed on a scatterplot of CD8*β *against CD4 for the CD3^+ ^population**. The red and purple clusters at the upper right correspond to the CD3^+^CD4^+^CD8*β*^+ ^cell population, indicative of the GvHD.

**Figure 5:**
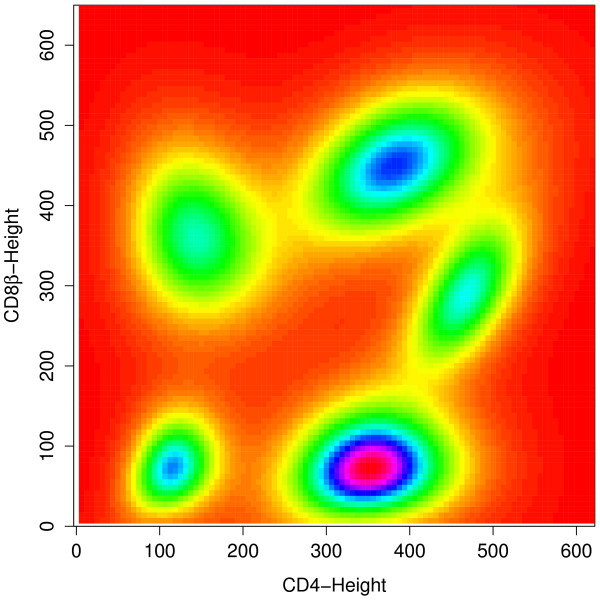
**An image plot of CD8*β *against CD4 for the CD3^+ ^population**. The five clusters corresponding to the CD3^+ ^population shown in Figure 5 can also be identified clearly on this image plot.

The BIC curve remains relatively flat beyond 11 clusters (Figure 3), suggesting that the model with 11 clusters provides a good fit. Figure 4 shows a contour plot superimposed on a scatterplot of CD8*β *against CD4 for the sub-population of CD3-stained cells, which were selected based on a threshold obtained from a negative control sample [40]. We can easily identify from it the red and purple clusters at the upper right as the CD3^+^CD4^+^CD8*β*^+ ^cell population. A corresponding image plot is given by Figure 5. Also, see Additional file 5 for the code used to produce all the plots shown in this article.

The example above shows how an FCM analysis is conducted with the aid of **flowClust**. When the number of cell populations is not known in advance, and the BIC values are relatively close over a range of the possible number of clusters, the researcher may be presented with a set of possible solutions instead of a clear-cut single one. In such a case, the level of automation may be undermined as the researcher may need to select the best one based on his expertise. We acknowledge that more effort is needed to extend our proposed methodology towards a higher level of automation. Currently, we are working on an approach which successively merges the clusters in the solution as suggested by the BIC using some entropy criterion to give a more reasonable estimate of the number of clusters.

### Integration with flowCore

As introduced in the Background section, **flowClust **has been built in a way such that it is highly integrated with the **flowCore **package. The core function flowClust which performs the clustering operation may be replaced by a call to the constructor tmixFilter creating a filter  object similar to the ones used in other gating or filtering operations found in **flowCore **(e.g., rectangleGate, norm2Filter, kmeansFilter). As an example, the code

res1 <- flowClust(GvHD, varNames = c("FSC-H", "SSC-H"), K = 1:8)

used in the first-stage analysis of the GvHD data may be replaced by:

s1filter <- tmixFilter("lymphocyte", c("FSC-H", "SSC-H"), K = 1:8)

res1f <- filter(GvHD, s1filter)

The use of a dedicated tmixFilter-class object separates the task of specifying the settings (tmixFilter) from the actual filtering operation (filter), facilitating the common scenario in FCM gating analysis that filtering with the same settings is performed upon a large number of data files. The filter method returns a list object res1f with elements each of class tmixFilterResult, which directly extends the filterResult class defined in **flowCore**. Users may apply various subsetting operations defined for the filterResult class in a similar fashion on a tmixFilterResult object. For instance,

Subset(GvHD [, c("FSC-H", "SSC-H")], res1f[[4]])

outputs a flowFrame that is the subset of the GvHD data upon the removal of outliers, consisting of the two selected parameters, FSC-H and SSC-H, only. Another example is given by the split method introduced earlier in this section.

We realize that occasionally a researcher may opt to combine the use of **flowClust **with filtering operations in **flowCore **to define the whole sequence of an FCM gating analysis. To enable the exchange of results between the two packages, filters created by tmixFilter may be treated like those from **flowCore**; users of **flowCore **will find that filter operators, namely, &, |, ! and %subset%, also work in the **flowClust **package. For instance, suppose the researcher is interested in clustering the CD3^+ ^cell population which he defines by constructing an interval gate with the lower end-point at 270 on the CD3 parameter. He may use the following code to perform the analysis:

rectGate <- rectangleGate(filterId="CD3+", "FL3-H" =c(270, Inf))

s2filter <- tmixFilter("s2filter", c("FL1-H", "FL2-H", "FL3-H", "FL4-H"), K = 5)

res2f <- filter(GvHD2$lymphocyte, s2filter %subset% rectGate)

The constructors rectangleGate and tmixFilter create two filter objects storing the settings of the interval gate and flowClust, respectively. When the last line of code is run, the interval gate will first be applied to the GvHD data. flowClust is then performed on a subset of the GvHD data contained by the interval gate.

## Conclusion

**flowClust **is an **R **package dedicated to FCM gating analysis, addressing the increasing demand for software capable of processing and analyzing the voluminous amount of FCM data efficiently via an objective, reproducible and automated means. The package implements a statistical clustering approach using multivariate *t *mixture models with the Box-Cox transformation [30], and provides tools to summarize and visualize results of the analysis. The statistical model underlying **flowClust **extends the one originally proposed in [30]. The extensions have included modeling options allowing for a cluster-specific estimation of the Box-Cox parameter *λ *and the degrees of freedom parameter *ν*. The package contributes to the cytometry community by offering an efficient, automated analysis platform which facilitates the active, ongoing technological advancement.

## Availability and requirements

Project name: flowClust

Project homepage: 

Operating systems: Platform independent

Programming language: C, R

Other requirements: GSL, R, Bioconductor

License: Artistic 2.0

Any restrictions to use by non-academics: **flowClust **depends on the **mclust **software, the use of which needs to abide by the terms stated in .

## Authors' contributions

KL and RG developed the methodology and software, and performed the analyses. FH participated in the development of the software. RRB and RG conceived of the study, and participated in its design and coordination. FH, RRB and RG helped KL draft the manuscript. All authors read and approved the final manuscript.

## Supplementary Material

Additional file 1 **A copy of the flowClust package**. The zip file contains the source code of the **flowClust **package (version 2.2.0) as a gzipped tarball for direct installation into R from a command-line interface. This current release is also available from Bioconductor at . Click here for file

Additional file 2 **A copy of the GvHD data file used in this article**. The zip file contains the data file in FCS format used in the GvHD analysis. Interested readers may go to  for a complete set of data files for the GvHD study [40]. Click here for file

Additional file 3 **A graph with two BIC curves corresponding to the settings with a common λ and cluster-specific λ respectively for the first-stage cluster analysis**. Little difference in the BIC values between the two settings is observed. In accordance with the principle of parsimony which favors a simpler model, we opt for the default setting here. Click here for file

Additional file 4 **Result summary of the first-stage analysis with four clusters of the GvHD data**. The rule used to identify outliers is 95% quantile. 133 points (1.03%) are called outliers. Click here for file

Additional file 5 **Code to produce the plots in this article**. R code to produce the plots in the GvHD analysis. Click here for file
