# Supplementary figures and images for: flowClust: a Bioconductor package for automated gating of flow cytometry data
Source: BMC Bioinformatics. 2009 May 14;10:145. doi: 10.1186/1471-2105-10-145 (PMC2701419; doi:10.1186/1471-2105-10-145)

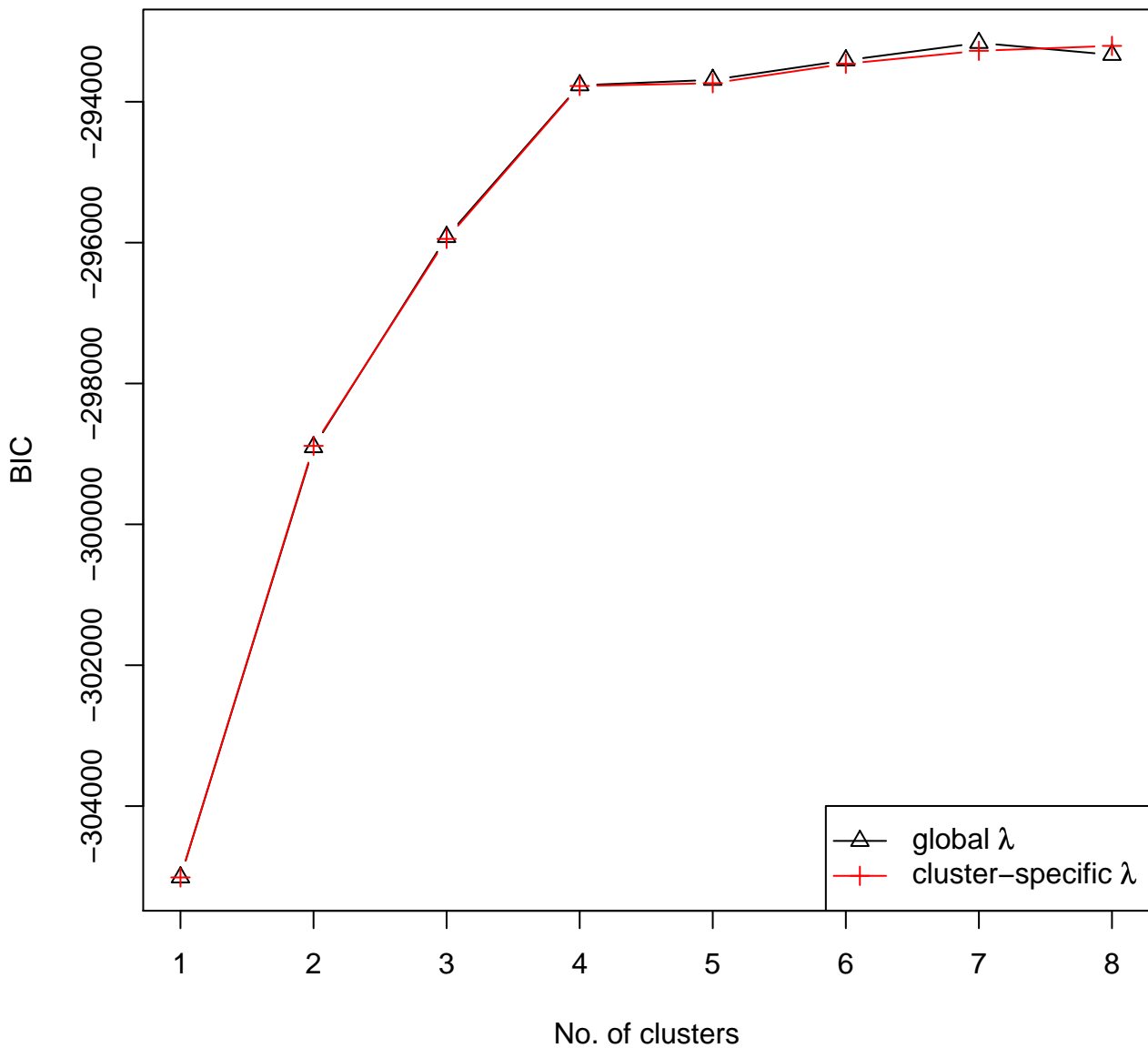

Supplement: Additional file 3 — A graph with two BIC curves corresponding to the settings with a common λ and cluster-specific λ respectively for the first-stage cluster analysis. Little difference in the BIC values between the two settings is observed. In accordance with the principle of parsimony which favors a simpler model, we opt for the default setting here. [file 1471-2105-10-145-S3.pdf]
