# Supplementary material for: flowClust: a Bioconductor package for automated gating of flow cytometry data
Source: BMC Bioinformatics. 2009 May 14;10:145. doi: 10.1186/1471-2105-10-145 (PMC2701419; doi:10.1186/1471-2105-10-145)
Supplement: Additional file 1 — A copy of the flowClust package. The zip file contains the source code of the flowClust package (version 2.2.0) as a gzipped tarball for direct installation into R from a command-line interface. This current release is also available from Bioconductor at . [file 1471-2105-10-145-S1.zip › flowClust_2.2.0.tar.gz!/flowClust/inst/doc/flowClust.pdf]

Robust Model-based Clustering of Flow Cytometry Data
The flowClust package

Raphael Gottardo, Kenneth Lo

April 21, 2009

raph@stat.ubc.ca, c.lo@stat.ubc.ca

Contents

1 Licensing **2**

2 Overview **2**

3 Installation **2**

4 Example: clustering of the Rituximab dataset **3**

4.1 The core function 3

4.2 Visualization of clustering results 7

4.3 Integration with flowCore 11

1 Licensing

Under the Artistic License, you are free to use and redistribute this software. However, we ask you to cite the following paper if you use this software for publication.

> Lo, K., Brinkman, R. R., and Gottardo, R. (2008) Automated Gating of Flow Cytometry Data via Robust Model-based Clustering. Cytometry A 73:321–332.

2 Overview

We apply a robust model-based clustering approach proposed by Lo et al. (2008) to identify cell populations in flow cytometry data. The proposed approach is based on multivariate t mixture models with the Box-Cox transformation. This approach generalizes Gaussian mixture models by modeling outliers using t distributions and allowing for clusters taking non-ellipsoidal shapes upon proper data transformation. Parameter estimation is carried out using an Expectation-Maximization (EM) algorithm which simultaneously handles outlier identification and transformation selection. Please refer to Lo et al. (2008) for more details. Whereas this research primarily arose to automate the gating process in flow cytometry, the proposed clustering approach is a general methodology which – and also this package – can be applied to cluster analyses in many other fields.

This `flowClust` package consists of a core function to implement the aforementioned clustering methodology. Its source code is built in C for optimal utilization of system resources. Several plotting functions or methods are available to users for visualizing various features of the clustering results including the cluster assignment, outliers, and the size and shape of the clusters formed. The fitted mixture model may be projected onto any one/two dimensions and displayed by means of a contour or image plot. Currently, the `flowClust` package provides two options for estimating the number of mixture components when it is unknown, namely, the Bayesian Information Criterion (BIC) and the Integrated Completed Likelihood (ICL).

`flowClust` is built in a way such that it is highly integrated with `flowCore`, the core package for flow cytometry that provides data structures and basic functionalities to process cytometry data. Please read Section 4.3 below for details about the actual implementations.

3 Installation

To build the `flowClust` package from source, make sure that the following is present in your system:

- GNU Scientific Library (GSL)

- Basic Linear Algebra Subprograms (BLAS)
- a C compiler

GSL can be downloaded at http://www.gnu.org/software/gsl/. In addition, the package uses BLAS to perform basic vector and matrix operations. Please go to http://www.netlib.org/blas/faq.html#5 for a list of optimized BLAS libraries for a variety of computer architectures. For instance, Mac users may use the built-in vecLib framework, while users of Intel machines may use the Math Kernel Library (MKL). A C compiler is needed to build the package as the core of the `flowClust` function is coded in C.

For the package to be installed properly you might have to type the following command before installation:

```
export LD_LIBRARY_PATH='/path/to/GSL/:/path/to/BLAS/':$LD_LIBRARY_PATH
```

which will tell **R** where your GSL and BLAS libraries (see below for more details about BLAS libraries) are. Note that this might have already been configured on your system, so you might not have to do so. In case you need to do it, you might consider copying and pasting the line in your `.bashrc` so that you do not have to do it every time.

Now you are ready to install the package:

```
R CMD INSTALL flowClust_x.y.z.tar.gz
```

The package will look for a BLAS library on your system, and by default it will choose gslcblas, which is not optimized for your system. To use an optimized BLAS library, you can use the `--with-blas` argument which will be passed to the `configure.ac` file. For example, on a Mac with vecLib pre-installed the package may be installed via:

```
R CMD INSTALL flowClust_x.y.z.tar.gz --configure-args="--with-blas='-
framework vecLib'"
```

On a 64-bit Intel machine which has MKL as the optimized BLAS library, the command may look like:

```
R CMD INSTALL flowClust_x.y.z.tar.gz --configure-args="--with-blas='-
L/usr/local/mkl/lib/em64t/ -lmkl -lguide -lpthread'"
```

where `/usr/local/mkl/lib/em64t/` is the path to MKL.

If you prefer to install a prebuilt binary, you need GSL for successful installation.

4 Example: clustering of the Rituximab dataset

4.1 The core function

To demonstrate the functionality we use a flow cytometry dataset from a drug-screening project to identify agents that would enhance the anti-lymphoma ac-

tivity of Rituximab, a therapeutic monoclonal antibody. The dataset is an object of class `flowFrame`; it consists of eight variables, among them only the two scattering variables (`FSC.H`, `SSC.H`) and two channels for the fluorochrome measurements (`FL1.H`, `FL3.H`) are of interest in this experiment. Note that, apart from a typical matrix object, the `flowClust` package may directly take a `flowFrame` object, a standard **R** implementation of an "FCS" file which may be returned from the `read.FCS` function in the `flowCore` package, as data input. The following code performs an analysis with one cluster using the two scattering variables:

```
> library(flowClust)
```

```
> data(rituximab)
> summary(rituximab)
```

```
         FSC.H  SSC.H FL1.H FL2.H  FL3.H   FL1.A FL1.W Time
Min.      59.0   11.0   0.0   0.0    1.0    0.00   0.0    2
1st Qu.  178.0  130.0 197.0  55.0  150.0    0.00   0.0  140
Median   249.0  199.0 244.0 116.0  203.0    0.00   0.0  285
Mean     287.1  251.8 349.2 126.4  258.3   73.46  17.6  294
3rd Qu.  331.0  307.0 445.0 185.0  315.0    8.00   0.0  451
Max.    1023.0 1023.0 974.0 705.0 1023.0 1023.00 444.0  598
```

```
> res1 <- flowClust(rituximab, varNames = c("FSC.H", "SSC.H"),
+     K = 1, B = 100)
```

`B` is the maximum number of EM iterations; for demonstration purpose here we set a small value for `B`. The main purpose of performing a cluster analysis with one cluster here is to identify outliers, which will be removed from the subsequent analysis.

Next, we would like to proceed with an analysis using the two channels for the fluorochrome measurements on the cells selected at the first stage. The following code performs the analysis with the number of clusters being fixed from one to six respectively:

```
> rituximab2 <- rituximab[rituximab %in% res1, ]
> res2 <- flowClust(rituximab2, varNames = c("FL1.H", "FL3.H"),
+     K = 1:6, B = 100)
```

We select the best model based on the Bayesian Information Criterion (BIC). The BIC value of a fitted model can be retrieved through the `criterion` method. By inspection, the BIC values stay relatively flat beyond three clusters. We therefore choose the model with three clusters and print a summary of the corresponding clustering result:

```
> criterion(res2, "BIC")
```

```
[1] -34167.12 -33103.87 -33080.88 -33056.55 -33055.49 -32978.00
```

```
> summary(res2[[3]])

** Experiment Information **
Experiment name: Flow Experiment
Variables used: FL1.H FL3.H
** Clustering Summary **
Number of clusters: 3
Proportions: 0.2658702 0.5091045 0.2250253
** Transformation Parameter **
lambda: 0.4312673
** Information Criteria **
Log likelihood: -16475.41
BIC: -33080.88
ICL: -34180.67
** Data Quality **
Number of points filtered from above: 0 (0%)
Number of points filtered from below: 0 (0%)
Rule of identifying outliers: 90% quantile
Number of outliers: 96 (6.99%)
Uncertainty summary:
     Min.   1st Qu.    Median      Mean   3rd Qu.      Max.
0.0005699 0.0153000 0.1669000 0.2019000 0.3738000 0.5804000
```

The summary states that the rule used to identify outliers is "`90% quantile`", which means that a point outside the 90% quantile region of the cluster to which it is assigned will be called an outlier. To specify a different rule, we make use of the `ruleOutliers` replacement method. The example below applies the more conservative "`95% quantile`" rule to identify outliers:

```
> ruleOutliers(res2[[3]]) <- list(level = 0.95)

Rule of identifying outliers: 95% quantile

> summary(res2[[3]])

** Experiment Information **
Experiment name: Flow Experiment
Variables used: FL1.H FL3.H
** Clustering Summary **
Number of clusters: 3
Proportions: 0.2658702 0.5091045 0.2250253
** Transformation Parameter **
lambda: 0.4312673
** Information Criteria **
Log likelihood: -16475.41
BIC: -33080.88
ICL: -34180.67
```

```
** Data Quality **
Number of points filtered from above: 0 (0%)
Number of points filtered from below: 0 (0%)
Rule of identifying outliers: 95% quantile
Number of outliers: 35 (2.55%)
Uncertainty summary:
     Min.   1st Qu.    Median      Mean   3rd Qu.      Max.
0.0005699 0.0153000 0.1669000 0.2019000 0.3738000 0.5804000
```

We can also combine the rule set by the `z.cutoff` argument to identify outliers. Suppose we would like to assign an observation to a cluster only if the associated posterior probability is greater than 0.5. We can add this rule with the following command:

```
> ruleOutliers(res2[[3]]) <- list(z.cutoff = 0.6)
```

```
Rule of identifying outliers: 95% quantile,
                              probability of  assignment < 0.6
```

```
> summary(res2[[3]])
```

```
** Experiment Information **
Experiment name: Flow Experiment
Variables used: FL1.H FL3.H
** Clustering Summary **
Number of clusters: 3
Proportions: 0.2658702 0.5091045 0.2250253
** Transformation Parameter **
lambda: 0.4312673
** Information Criteria **
Log likelihood: -16475.41
BIC: -33080.88
ICL: -34180.67
** Data Quality **
Number of points filtered from above: 0 (0%)
Number of points filtered from below: 0 (0%)
Rule of identifying outliers: 95% quantile,
                              probability of  assignment < 0.6
Number of outliers: 317 (23.07%)
Uncertainty summary:
     Min.   1st Qu.    Median      Mean   3rd Qu.      Max.
0.0005699 0.0153000 0.1669000 0.2019000 0.3738000 0.5804000
```

This time more points are called outliers. Note that such a change of the rule will not incur a change of the model-fitting process. The information about which points are called outliers is conveyed through the "`flagOutliers`" slot, a logical vector in which the positions of `TRUE` correspond to points being called outliers.

By default, when 10 or more points accumulate on the upper/lower boundary of any dimension, the `flowClust` function will filter those points. To change the threshold count from default (i.e., 10), users may specify `max.count` and `min.count` when running `flowClust`. To suppress filtering at the upper and/or the lower boundaries, set `max.count` and/or `min.count` as -1. We can also use the `max` and `min` arguments to control filtering of points, but from a different perspective. For instance, if we are only interested in cells which have a `FL1.H` measurement within (0, 400) and `FL3.H` within (0, 800), we may use the following code to perform the cluster analysis:

```
> flowClust(rituximab2, varNames = c("FL1.H", "FL3.H"), K = 2,
+     B = 100, min = c(0, 0), max = c(400, 800))
```

4.2 Visualization of clustering results

Information like the cluster assignment, cluster shape and outliers may be visualized by calling the `plot` method to make a scatterplot:

```
> plot(res2[[3]], data = rituximab2, level = 0.8, z.cutoff = 0)
```

```
Rule of identifying outliers: 80% quantile
```

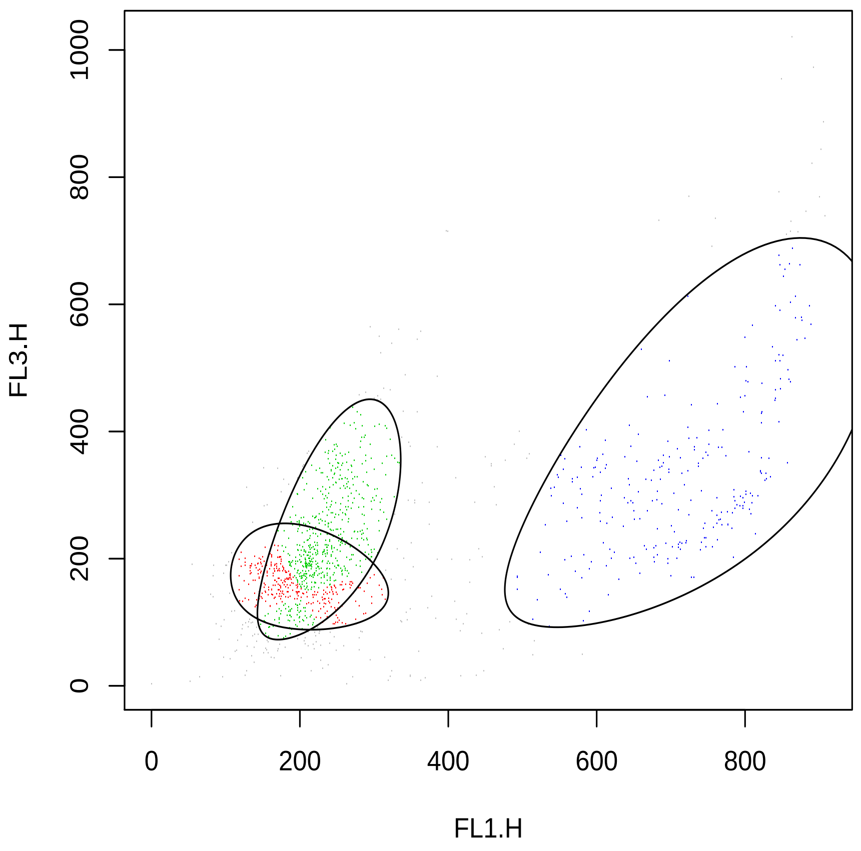

The `level` and/or `z.cutoff` arguments are needed when we want to apply a rule different from that stored in the `ruleOutliers` slot of the `flowClust` object to identify outliers.

To look for densely populated regions, a contour/image plot can be made:

```
> res2.den <- density(res2[[3]], data = rituximab2)
> plot(res2.den)
```

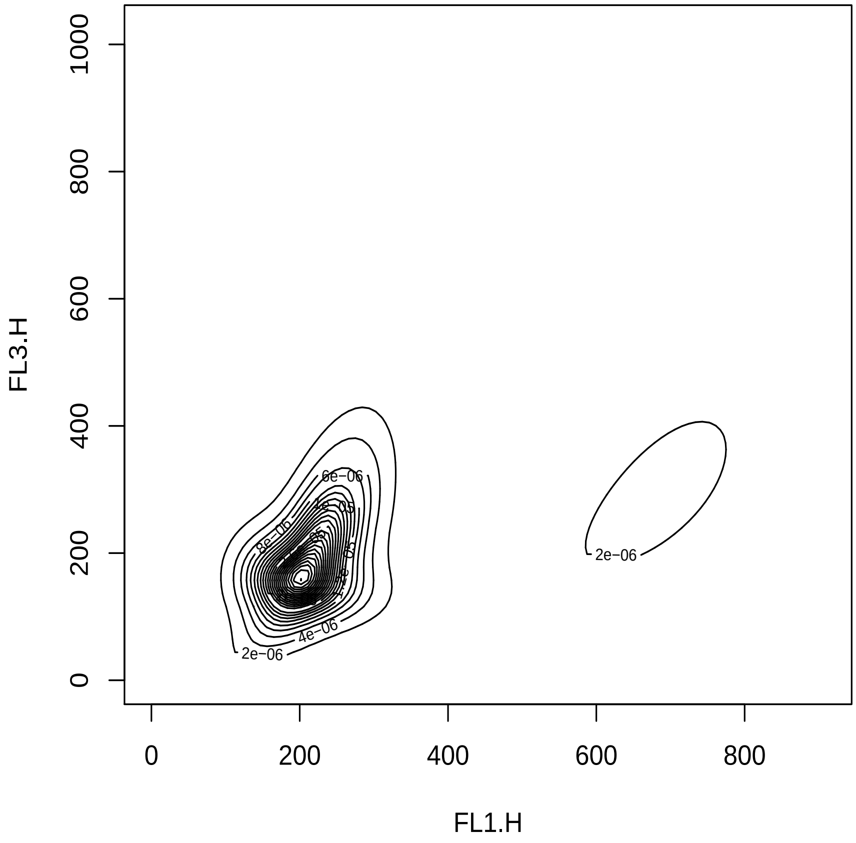

```
> plot(res2.den, type = "image")
```

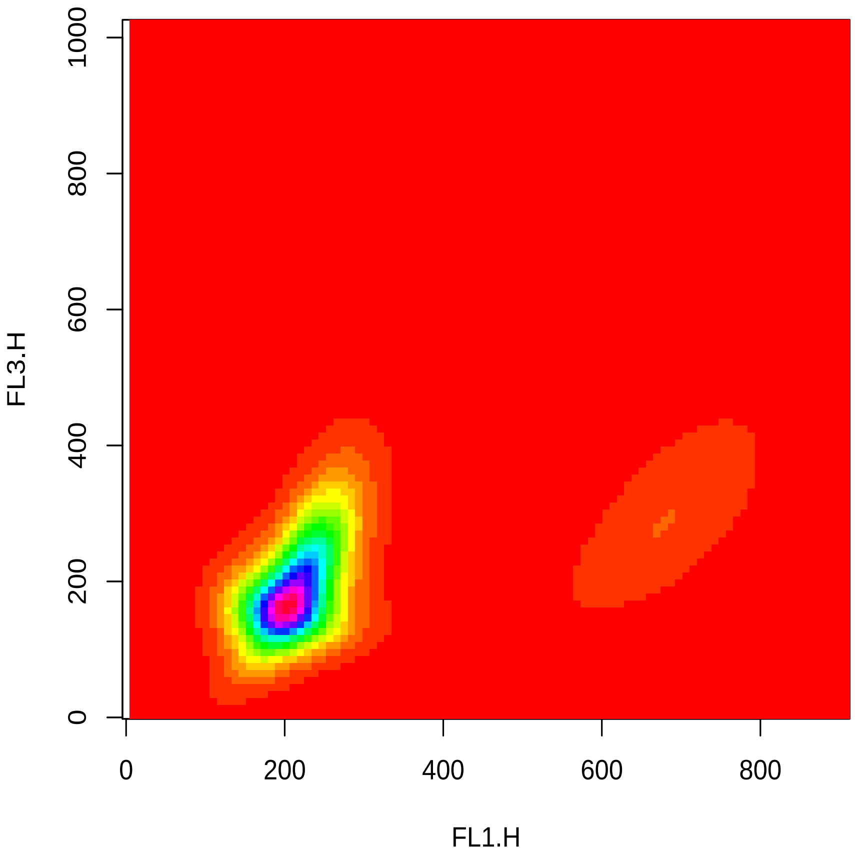

When we want to examine how the fitted model and/or the data is distributed along one chosen dimension, we can use the `hist` method:

```
> hist(res2[[3]], data = rituximab2, subset = "FL1.H")
```

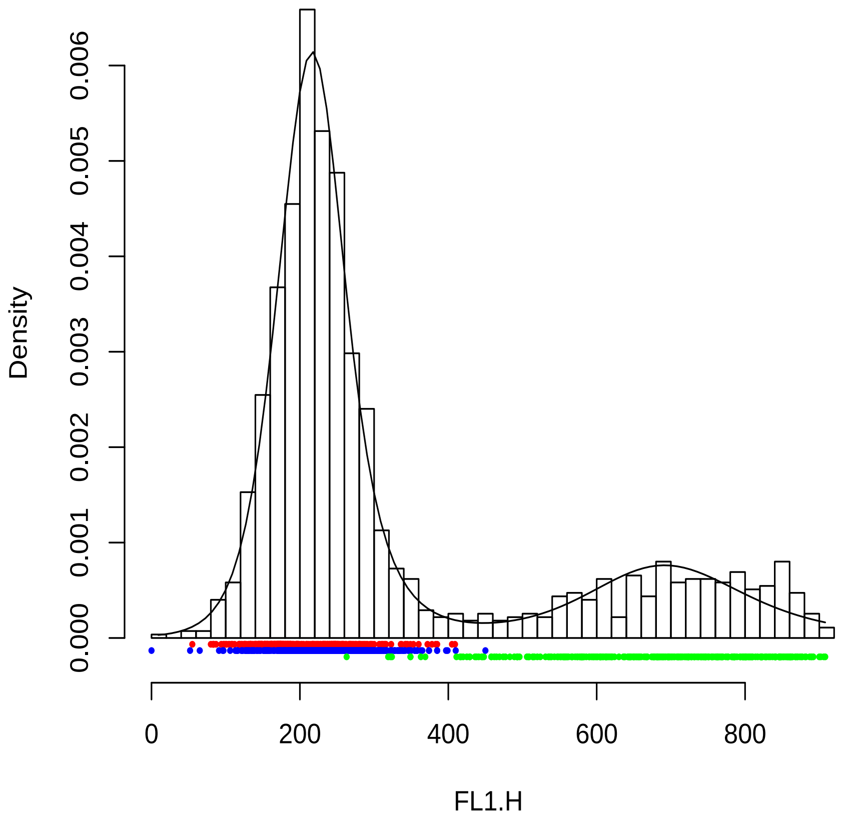

The `subset` argument may also take a numeric value:

```
> hist(res2[[3]], data = rituximab2, subset = 1)
```

Since `FL1.H` is the first element of `res2[[3]]@varNames`, this line produces exactly the same histogram as the one generated by the line taking `subset="FL1.H"`. Likewise, the `subset` argument of both `plot` methods accepts either a numeric or a character vector to specify which two variables are to be shown on the plot.

4.3 Integration with flowCore

As mentioned in the Overview, effort has been made to make this package highly integrate with the `flowCore` package. Users will find that most methods defined in `flowCore` also work well in the context of `flowClust`.

The very first step of integration is to replace the `flowClust` function with the `tmixFilter-filter` method pair. The aim is to wrap the clustering methodology in a filtering operation like those found in `flowCore`. The `tmixFilter` function creates a `filter` object to store all settings required for filtering (i.e., clustering). The object created is then passed to the actual filtering operation implemented by the `filter` method. The use of the `tmixFilter-filter` method pair separates the task of specifying the settings from the actual filtering operation, which facilitates a usual scenario in which filtering with the same settings is performed upon a set of data files.

As an example, the filtering operation that resembles the second-stage clustering using `FL1.H` and `FL3.H` with 3 clusters (see Section 4.1) is implemented by the following code:

```
> s2filter <- tmixFilter("s2filter", c("FL1.H", "FL3.H"), K = 3,
+     B = 100)
> res2f <- filter(rituximab2, s2filter)
```

The object `res2f` is of class `tmixFilterResult`, which extends the `multiple-FilterResult` class created by `flowCore`. Users may apply various subsetting operations defined for the `multipleFilterResult` class in a similar manner on a `tmixFilterResult` object:

```
> Subset(rituximab2, res2f)

flowFrame object 'A02'
with 1267 cells and 8 observables:
     name              desc range
$P1 FSC.H        FSC-Height  1024
$P2 SSC.H       Side Scatter  1024
$P3 FL1.H     Anti-BrdU FITC  1024
$P4 FL2.H               <NA>  1024
$P5 FL3.H              7 AAD  1024
$P6 FL1.A               <NA>  1024
$P7 FL1.W               <NA>  1024
$P8  Time Time (204.80 sec.)  1024
135 keywords are stored in the 'descripton' slot

> split(rituximab2, res2f, population = list(sc1 = 1:2, sc2 = 3))

$sc1
flowFrame object 'A02 (1,2)'
with 976 cells and 8 observables:
     name              desc range
$P1 FSC.H        FSC-Height  1024
$P2 SSC.H       Side Scatter  1024
$P3 FL1.H     Anti-BrdU FITC  1024
$P4 FL2.H               <NA>  1024
$P5 FL3.H              7 AAD  1024
```

```
$P6 FL1.A               <NA>  1024
$P7 FL1.W               <NA>  1024
$P8  Time Time (204.80 sec.)  1024
3 keywords are stored in the 'descripton' slot

$sc2
flowFrame object 'A02 (3)'
with 291 cells and 8 observables:
     name               desc range
$P1 FSC.H         FSC-Height  1024
$P2 SSC.H       Side Scatter  1024
$P3 FL1.H     Anti-BrdU FITC  1024
$P4 FL2.H               <NA>  1024
$P5 FL3.H              7 AAD  1024
$P6 FL1.A               <NA>  1024
$P7 FL1.W               <NA>  1024
$P8  Time Time (204.80 sec.)  1024
136 keywords are stored in the 'descripton' slot
```

The `Subset` method above returns a `flowFrame` object consisting of observations within the data-driven filter constructed. The `split` method splits the data into 2 populations upon the removal of outliers: the first population is formed by observations assigned to clusters 1 and 2 constructed by the filter, and the other population consists of observations assigned to cluster 3. The two populations are returned as two `flowFrame` objects separately, which are stored inside a list and labelled with `sc1` and `sc2` respectively.

The `%in%` operator is also defined for a `tmixFilterResult` object, which will return a logical vector in which a `TRUE` value means that the corresponding observation is accepted by the filter. In fact, the implementation of the `Subset` method needs to call `%in%`.

The object returned by `tmixFilter` is of class `tmixFilter`, which extends the `filter` class in `flowCore`. Various operators, namely, `&`, `|`, `!` and `%subset%`, which have been constructed for `filter` objects in `flowCore`, also produce expected outcomes when applied to a `tmixFilter` object. For example, to perform clustering on a subset evaluated by a rectangle gate, we may apply the following code:

```
> rectGate <- rectangleGate(filterId = "rectRegion", FL1.H = c(0,
+     400), FL3.H = c(0, 800))
> MBCfilter <- tmixFilter("MBCfilter", c("FL1.H", "FL3.H"), K = 2,
+     B = 100)
> filter(rituximab2, MBCfilter %subset% rectGate)

A filterResult produced by the filter named 'MBCfilter in rectRegion'
```
